# The Role of the NIS (SLC5A5) Gene in Papillary Thyroid Cancer: A Systematic Review

**DOI:** 10.1155/2018/9128754

**Published:** 2018-11-14

**Authors:** Rafael Martins de Morais, Alaor Barra Sobrinho, Calliandra Maria de Souza Silva, Jamila Reis de Oliveira, Izabel Cristina Rodrigues da Silva, Otávio de Toledo Nóbrega

**Affiliations:** ^1^Post-Graduate in Medical Sciences Program, University of Brasília, Brasília, DF, Brazil; ^2^Imagens Médicas de Brasília, Brasília, DF, Brazil; ^3^Institute of Biological Sciences, University of Brasília, Brasília, DF, Brazil; ^4^Faculty of Ceilândia, University of Brasília, Brasília, DF, Brazil

## Abstract

Papillary thyroid cancer (PTC) is the most common thyroid malignancy. Genetic and epigenetic alterations play a decisive role in the onset of several human neoplasms. Mutations and polymorphisms are two frequent genetic alterations. Located on chromosome 19 (19p13.11), the NIS SLC5A5 (solute carrier family 5 member 5) gene encodes a highly specialized and efficient 80–90 kDa transmembrane glycoprotein that mediates active transport of iodide from the bloodstream into the follicular cells. Given the highly significant role of NIS in the physiology and the cancer pathogenesis process, this paper's objective is to provide a comprehensive assessment of the associations between NIS gene and protein with papillary thyroid cancer.

## 1. Introduction

The thyroid gland is one of the largest glands in the human body. Located anterolaterally to the trachea and larynx, it consists of two lobes connected by an isthmus. It is composed histologically of two main types of parenchymal cells: the follicular ones, where there is a higher concentration of iodine and the production of thyroid hormones (HT) T3 and T4, and the parafollicular cells, in which the hormone calcitonin is produced. There are reports that from the follicular cells emerge the well-differentiated cancers (papillary and follicular), whereas in the parafollicular region originate medullary carcinoma [1].

The incidence of thyroid cancer is increasing among men and women, driven by the papillary subtype, representing approximately 90% of cases. When comparing the incidence by gender, the rates are higher in women than in men, of which the greater representativeness is in whites, besides increasing with age [2].

Mortality rates are highest in the Melanesian region, in some regions of Africa, as well as in countries with lower levels of human development, although the highest rates of incidence are reported in some European countries and North America. Nevertheless, the highest overall incidence is in the Republic of Korea, where thyroid cancer is the most common cancer among women [3].

In Brazil, for the years 2016 and 2017, 1090 new cases were expected for males and 5870 for females, with an estimated risk of 0.97 cases per 100,000 males and 5.62 cases per 100,000 females [4]. In the United States, the results showed that from 1975 to 2014, the incidence almost tripled, from 4.9 to 14.3 per 100,000 individuals. Substantially all of the increase was attributed to papillary thyroid cancer (PTC) (from 3.4 to 12.5 per 100,000). However, it was concluded that there is no epidemic of the disease in the United States but rather a diagnostic epidemic [5].

PTC is the most common endocrine malignancy accounting for approximately 80–85% of cases [1]. Radiation exposure, age, female gender, and family history are some risk factors that increase the incidence of well-differentiated thyroid cancer. Epidemiological studies have demonstrated an increased risk of four to ten times greater in individuals with first-degree relatives with this neoplasm [6].

A retrospective case-control study with 255 patients evaluated possible associations between single nucleotide polymorphisms (SNPs) in the ATM, XRCC1, TP53, XRCC3, and MTF1 genes with associated PTC risk. The results showed that SNPs in exon 39 of the ATM gene and the exon 10 of the XRCC1 gene may be the markers of protection in adults' PTC, whereas, in the SNPs of ATM, IVS22-77 and TP53 genes at codon 72 may be associated with the risk of PTC development in nonirradiated and irradiated subjects [7].

Activation of the MAPK and PI3K/AKT signaling pathways appears to be crucial for the initiation and progression of thyroid cancer [8]. The BRAF and RAS genes are also commonly found in TCs, as well as the changes involving RET/PTC and PAX8/PPAR-*γ* chromosomal rearrangements [9, 10].

A genomic association study of nonmedullary thyroid cancer included 3.001 patients and 287.550 controls from five study groups of European descent. The results showed five new loci (all with combined *P* < 3 × 10^−8^): 1q42.2 (rs12129938 in PCNXL2), 3q26.2 (rs6793295 a missense mutation in LRCC34 next to TERC), 5q22.1 (rs73227498 between NREP and EPB41L4A), 10q24.33 (rs7902587 close to OBFC1), and two variants independently associated with 15q22.33 (rs2289261 and rs56062135, both in SMAD3) [11].

Another paper describes the genomic situation of 496 PTCS: a low frequency of somatic alterations (relative to other carcinomas) and an extension of the set of known PTC driver alterations to include EIF1AX, PPM1D, and CHEK2 and diverse gene fusions. These grouped analyses of gene expression, genomic variants, and methylations have demonstrated that different groups lead to different pathologies with distinct characteristics of signaling and differentiation [12].

Located on chromosome 19 (19p13.11), the NIS SLC5A5 (solute carrier family 5 member 5) gene encodes a highly specialized and efficient 80–90 kDa transmembrane glycoprotein that mediates active transport of iodide from the bloodstream into the follicular cells. This transport occurs via the sodium gradient generated by the Na+/K+-ATPase pump, transporting two sodium cations to an iodide anion as the crucial first step in the biosynthesis of thyroid hormones [13, 14]. NIS (SLC5A5) is also present in other tissues, such as gastric mucosa, choroid plexus, ciliary body of the eye, salivary and lacrimal glands, pulmonary airway, placenta, and mammary glands in the lactation period [15, 16]. The activity of NIS (SLC5A5) is mainly regulated by the thyroid-stimulating hormone (TSH), which consequently participates in the regulation of iodine uptake by follicular cells mediated by cAMP [15].

Given the highly significant role of NIS (SLC5A5) in the physiology and the cancer pathogenesis process, this paper's objective is to provide a comprehensive assessment of the associations between the NIS (SLC5A5) gene and protein with papillary thyroid cancer.

## 2. Materials and Methods

### 2.1. Search Strategy

**Figure 1:**
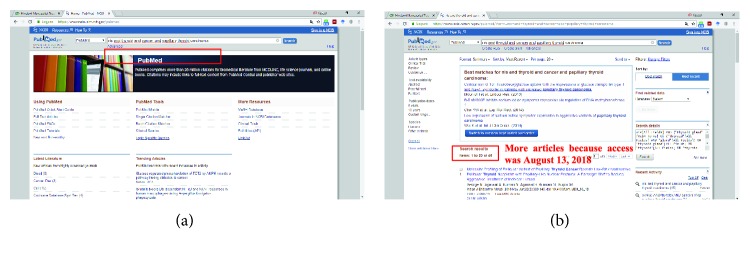


The literature search was carried out through the PubMed portal, on December 27, 2017, for all types of studies which may indicate the relationship between the NIS (SLC5A5) gene and susceptibility to papillary thyroid cancer. The following search terms were used: “NIS,” “thyroid,” “cancer,” and “papillary thyroid carcinoma.” The retrieved articles were selected by reviewers to identify search-related studies, as viewed in (Figure 1).

### 2.2. Filters and Inclusion and Exclusion Criteria

There were no restrictions regarding language, year, species, and types of studies in the bibliographic research. The inclusion criteria were as follows: (1) the study should relate the NIS gene to papillary thyroid cancer, even if associated with other comorbidities and with other genes; (2) the study should provide data for analysis. The studies were excluded if (1) they were clinical case studies, (2) they were studies with insufficient available data, and (3) they presented data from other types of thyroid cancer or did not specify what type the patients presented.

### 2.3. Data Extraction

The data from the eligible studies were independently extracted according to the inclusion and exclusion criteria. For each study, the following characteristics were collected: authors, study title, objective, year of publication, the country in which it was published, sample size, laboratory methodology, research results, and statistical values.

## 3. Results and Discussion

The systematic search found thirty-eight publications, between 1996 and 2017, of which nineteen met the inclusion criteria and are represented chronologically in Table 1.

**Figure 2:**
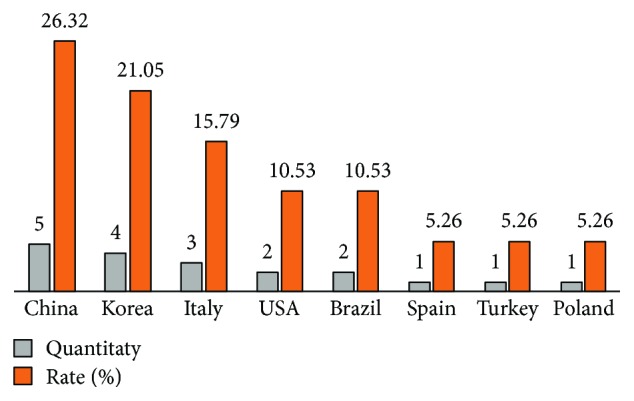
Quantities and rates of publications by country of selected publications using the inclusion criteria.

Of the articles selected, five were published in China, four in the Republic of Korea, three in Italy, two in the United States, and two in Brazil, followed by Spain, Turkey, and Poland with one each (Figure 2).

### 3.1. Expression, Localization, and Regulation of NIS (SLC5A5)

For years, lack of information on NIS (SLC5A5) built a significant gap in the understanding of thyroid pathophysiology. No molecular information of this gene was available until the year 1996 when Smanik et al. [35] reported the sequence of 1929 nucleotides that encode the NIS (SLC5A5) protein with 643 amino acids, thus proposing possible lines of investigations about its regulatory mechanisms and factors related to thyroid diseases [13, 35]. Since then, several studies have demonstrated the importance of the gene in the hormonal synthesis, iodine therapy, and physiology of the thyroid gland.

The NIS (SLC5A5) gene has also been reported in other tissues, such as in human testes, an important factor in predicting the accumulation of radioiodine in the gonads of men with thyroid cancer exposed to treatment, although the concentration represents levels 10 times lower than in thyroid tissues [36].

For the iodine transport which occurs from the bloodstream to the intracellular medium, the location of the NIS protein should be basolateral to the thyroid cell [37]. Wei et al. [26] demonstrated changes in protein expression of NIS in PTC, mainly located in the cytoplasm and rarely in the basolateral membrane when compared with healthy individuals; nonetheless, these data were not statistically significant (*P* = 0.675). Conversely, in tumors considered small (<1 cm), it presented a statistically significant difference (*P* = 0.037). Other locations have also been described, in another Chinese work, such as in the nucleus, in conventional PTC (*P* < 0.001) [26].

Another study demonstrated that NIS staining was localized almost exclusively to the plasma membrane in normal thyroid tissues but less intensely in tumor tissues [31]. Additionally, the positivity and the intensity of NIS expression were statistically higher between healthy and cancerous cells in a Korean study (*P* < 0.001) [18].

An article published in Italy in 2015 found that NIS expression levels in tumor tissue were lower than those in a healthy correspondent (*P* < 0.001). Furthermore, the expression of thyroid hormone receptor Beta (THR*β*) when compared to the NIS (SLC5A5) gene mRNA levels presented a statistically significant positive correlation (*r* = 0.3771, *P* = 0.0098) [30].

Another study demonstrated that NIS staining was localized almost exclusively on the plasma membrane in normal thyroid tissues but less intense in tumor tissues [31]. Other genes have also been identified as related to NIS (SLC5A5). The BRAFV600E alteration is the most common in PTC, with a worldwide prevalence of 29 to 83% [38]. Riesco-Eizaguirre et al. [17] sought to investigate the role of BRAFV600E and MEK-ERK (protein chain that communicates a signal from the receptor on the surface of the cell to DNA) in thyroid dedifferentiation. After immunohistochemical analysis, the NIS staining was negative or weakly positive and not localized to the membrane in most BRAFV600E versus BRAFwt samples (*P* = 0.005), and there is a limited role of the MEK-ERK pathway in this impairment. Likewise, in the Korean article, in the standard cell thyroid, the NIS staining was predominantly observed in the plasma membrane. However, the NIS presence was also located in the cytoplasm of cancer cells [18].

The functional expression of NIS, which allows the interiorization of iodine, was higher in patients with the BRAFV600E mutation (78.1%) than in those with the ancestral genotype (57.1%) (*P* = 0.047), while the nonfunctional NIS expression was lower in the BRAFV600E-positive mutation group (21.3%) than in the negative mutation group (42.9%) (*P* = 0.047) [33]. Therefore, it similar to those analyzed by Kleiman et al. [24], where the expression of NIS in the group of patients with TC (of which 86.5% were with PTC) with the BRAFV600E mutation was lower (0.27 ± 0.03) compared to BRAFwt (0.78 ± 0.09) (*P* < 0.01).

Given the regulation between BRAFwt gene expression and inhibition of NIS expression, the mutation in BRAFV600E did not inhibit NIS expression (*P* = 0.085) in PTC. However, in concomitance with Hashimoto's thyroiditis, there was less NIS expression after the adjustment for confounding factors (*P* = 0.046) [32]. However, for the first time, it was described in one paper, through an important epigenetic mechanism by histone deacetylation in the BRAFV600E gene, through the mechanism of chromatin compaction and the blocking of the promoters of the binding gene with transcription factors, resulting in the silencing of the NIS (SLC5A5) gene [39].

NIS expression was downregulated in BRAFV600E when expressed in normal primary thyrocytes isolated from tissues via lentiviral transduction (*P* < 0.01). By induction of methylation on the CpG island of the promoter region, BRAFV600E reduced NIS expression (29% in healthy cells versus 58% in PTC). Furthermore, real-time PCR showed that DNMT1 and NIS expression were inversely correlated with BRAFV600E (*P* < 0.01) [28].

The FoxP3 transcription factor is an intracellular molecule that suppresses antitumor responses that maintain the immune balance in host tissues. The high presence of FoxP3 in tissues is usually associated with an unfavorable outcome, so they are used to define prognosis and even new therapeutic targets [40]. Ma et al. [31] analyzed the NIS staining in the FoxP3-positive and FoxP3-negative groups. The positive group had a lower NIS staining rate (31.8%) than the negative one (66.7%) (*P* < 0.01). Furthermore, NIS transcript levels were significantly reduced in the tumor sample relative to normal tissue and in FoxP3-positive samples against FoxP3-negative samples (*P* < 0.001). The transforming growth factor-*β*1 (TGF-*β*1) was also explored. PTC cell samples were pretreated with TGF-*β*1 for 24 h. After analysis, NIS protein and transcript levels were found to decrease, reaching the minimum concentration of 10 ng/mL TGF-*β*1 when compared to the control (*P* < 0.01).

Smith et al. [19] sought to determine the methylation status of NIS (SLC5A5) promoter regions and to associate them with patients with papillary thyroid carcinoma. The results showed a higher methylation frequency in the malignant tissue when compared to the controls (*P* < 0.05), and in all controls, the promoter regions for NIS were not methylated. There was no statistical difference in the proportion of nodular metastasis between the methylated and unmethylated NIS samples (*P* = 0.17). The majority (57%) of the methylated NIS specimens presented nodular metastasis against 24% of the nonmethylated ones. Moreover, when tumor cells with adjacent healthy tissue were compared, methylation was only present in the pathological tissue.

NIS expression was also lower in BRAFV600E compared to the BRAFwt group in classic PTC (*P* < 0.0001) and PTC follicular variant (*P* = 0.0325) [20]. In a Brazilian study with 148 patients with differentiated thyroid cancer (DTC), of the thirteen that died, all tested negative for NIS in immunohistochemistry, in addition to weakly expressing mRNA of the gene [21]. In the same study, it was possible to observe that NIS expression was lower in PTC BRAFV600E (*P* = 0.0388), although it was not possible to identify individuals with worse prognosis after mRNA (*P* = 0.4037) and immunohistochemical analyses.

In order to analyze whether NIS expression was associated with the expression of BRAFV600E, Choi et al. [28] found that 65% of healthy thyroid follicle samples showed standard staining in the plasma membrane region. However, the membrane staining was reduced in PTC with a positive BRAFV600E. Moreover, 37% of PTC tissues exhibited diffuse cytoplasmic staining.

Unlike the natural physiology of the thyroid, NIS expression in the analyzed studies is impaired, corroborating the idea that alterations in the NIS SLC5A5 gene can lead to worse prognosis, due to the ineffectiveness of the radioiodine, though, in some studies, it is not possible to identify this through immunohistochemical analyzes (*P* = 0.4037) [21]. Patients with metastases in the region of the neck without uptake of ^131^I revealed a slight reduction in NIS transcripts when compared to primary patients (*P* = 0.05) [41]. NIS expression was higher in patients with TC well differentiated (0.67 ± 0.20) when compared to the less differentiated (0.36 ± 0.05) (*P* = 0.0001). Furthermore, there appears to be a competition of NIS SLC5A5 and GLUT-1 gene expression (*r* = −0.60, *P* < 0.01), positive correlation of NIS and TG (*r* = 0.52, *P* < 0.05), and NIS and pendrin (*r* = 0.77, *P* < 0.05) [27].

In most thyroid carcinomas, NIS mRNA levels are reduced when compared to adenomas and normal adjacent thyroid tissue [42]. In the study by Qin et al. [22], the positive expression of the NIS gene (70.3%) was statistically significant in patients with DTC (including PTC and FTC) when compared to follicular adenoma (*P* = 0.013). NIS gene expression was lower in PTC than in healthy tissue [41].

Normal thyroid cells express phosphodiesterase subtypes PDE4, PDE5, PDE7, and PDE8 [43]. Analyzing the PDE5 expression in surgical specimens of human papillary thyroid tumor tissues related to the gene BRAFV600E and NIS SLC5A5, Sponziello et al. concluded that all analyzed samples had higher levels of PDE5A expression compared to control samples. However, expression levels of NIS mRNA were decreased in PTC (*P* < 0.0001) [29].

### 3.2. The Role of NIS in Thyrocytes

The decay of ^131^I occurs by *β*-emission (606 keV) with 89% abundance of high energy *γ*-emission (364 keV). After its oral administration, it is rapidly absorbed in the gastrointestinal tract and then distributed to the thyroid and other organs. In the thyroid gland, the iodide is then internalized by the epithelial cells by active transport through the NIS protein. In addition to ^131^I, other radionuclides are also absorbed by thyroid cells and tissues, such as ^123^I, ^124^I, ^99m^Tc, ^111^In, ^18^F, ^11^C, and ^68^Ga [44].

The thyroid internalizes iodine through a series of metabolic steps which include some proteins such as pendrin (PD), thyroid peroxidase (TPO), and thyroglobulin (Tg), in addition to NIS [27].

The comparison of the uptake of FDG-F^18^ with the expression of GLUT1 and NIS in tumors of PTC patients undergoing thyroidectomy concluded that the maximum uptake pattern (SUV) in NIS-negative lesions was significantly higher than that in positive lesions (*P* = 0.006) [23].

Choi et al. found that there is no efficient iodide absorption when BRAFV600E is expressed in thyrocytes, resulting in inhibition of NIS expression in both PTC and isolated primary thyrocytes (*P* < 0.01) [28].

Positive regulation of NIS protein expression was observed in a Polish study in the K1 cell groups receiving lower (5 Gy) and higher (20 Gy) doses after 24 h with ^131^I (*P* < 0.05). However, after 72 h of culture without iodine, there was a reversal in regulation. Furthermore, changes in the 8-oxo-dG level were observed in cells treated with the lowest dose (5 Gy) when compared to the control group. The concentration increased only at the 96 h time point, indicating prolonged accumulation of DNA damage even after the completion of irradiation with ^131^I (*P* < 0.01) [34].

Several findings on the expression of genes in thyroid disease have shown that levels of mRNA-encoding NIS, thyrotropin receptor (TSH-R), thyroid peroxidase, and thyroglobulin (Tg), among other factors, are decreased in malignant tumors of the thyroid compared to controls [45].

## 4. Conclusion

The studies of genetic factors are of paramount importance for diagnosis, treatment, efficacy, and better prognosis in patients with papillary thyroid cancer. Although changes of these factors are not yet fully implemented and understood, identifying the presence of these alterations may allow early intervention of researchers and health professionals. The effectiveness of radioactive iodine (radioiodine) therapy depends not only on the role of the NIS SLC5A5 gene but also, conceivably, on the reduction of other molecules that regulate its intracellular metabolism.

## Figures and Tables

**Table 1:** Comparison of different studies of the role of NIS in papillary thyroid cancer.

Author	Title	Objective	Year	Country	Sample	Laboratorial test	Results	*P* value
Riesco-Eizaguirre et al. [17]	The oncogene BRAF V600E is associated with a high risk of recurrence and less differentiated papillary thyroid carcinoma due to the impairment of Na^+^/I^−^ targeting to the membrane	To investigate the role of BRAFV600E and MEK-ERK in the dedifferentiation of the thyroid	2006	Spain	*N* = 67	Immunohistochemistry	The NIS staining was either negative or weakly positive and not localized to the membrane.	*P* = 0.005
Lee et al. [18]	Relationship of sodium/iodide symporter expression with I131 whole body scan uptake between primary and metastatic lymph node papillary thyroid carcinomas	To evaluate the total and membranous NIS expression in the PTC papillary tissue, to associate it with the NIS expression between the PTC tissues of the primary and metastatic lymph node (LN), and to relate the NIS expression to the full-scan iodine-131 uptake between the primary tissues and metastatic lesions of the LN PTC	2007	Korea	*N* = 17	Immunohistochemistry	In cancer cells, the staining was located in the plasma membrane and cytoplasm.	NA
							In primary PTC, the positivity of NIS expression was higher between healthy cells and cancer cells.	*P* < 0.001
Smith et al. [19]	Methylation status of genes in papillary thyroid carcinoma	To determine the methylation status of the gene promoter regions and to associate with tumor factors or outcome measures among patients with papillary thyroid carcinoma	2007	USA	*N* = 32	MSP-PCR	The NIS gene was methylated in the malignant tissue more often than in controls. In all controls, the promoter regions for NIS were not methylated.	*P* < 0.05
							There was no statistical difference in the proportion of nodular metastasis between the methylated and unmethylated NIS samples.	*P* = 0.17
							NIS methylation in tumor cells was not accompanied by methylation in adjacent normal tissue.	NA
Mian et al. [41]	Molecular characteristics in papillary thyroid cancers (PTCs) with no 131I uptake	To characterize at the molecular level a subset of PTC without iodine-131 uptake	2008	Italy	*N* = 48	qPCR	NIS gene expression was lower in PTC than in normal tissue.	NA
							Patients with metastases in the neck region without 131I uptake revealed only a slight decrease in NIS transcripts compared to patients with the primary tumor.	*P* = 0.05
Oler and Cerutti [20]	High prevalence of BRAF mutation in a Brazilian cohort of patients with sporadic papillary thyroid carcinomas	To investigate the mutation status of BRAF and to correlate it with clinicopathological characteristics and expression of NIS and TSH-R	2009	Brazil	*N* = 120	qPCR	The expression of NIS was lower in the BRAFV600E group compared to the BRAFwt group in patients with classic PTC.	*P* < 0.0001
							The same also happened in the follicular variant.	*P* = 0.0325
Morari et al. [21]	Use of sodium iodide symporter expression in differentiated thyroid carcinomas	To investigate the use of NIS mRNA and protein expression as a diagnostic and/or prognosis marker in patients with differentiated thyroid cancer (TCD)	2011	Brazil	*N* = 148	Immunohistochemistry/qPCR	All 13 patients with DTC who died were NIS negative and had low mRNA expression.	NA
							The NIS expression was lower in PTC BRAFV600E.	*P* = 0.0388
							The data analyzed did not identify individuals with worse prognosis.	*P* = 0.4037
Qin et al. [22]	Correlation of clinicopathological features and expression of molecular markers With prognosis after 131I treatment of differentiated thyroid carcinoma	To identify molecular markers associated with patients with differentiated thyroid carcinoma (DTC) and to determine the existence of a correlation between clinicopathological characteristics or molecular markers with the result of iodine therapy	2012	China	*N* = 74	Immunohistochemistry	Positive NIS expression was different in patients with DTC.	*P* = 0.013
Moon et al. [23]	Comparison of 18F-fluorodeoxyglucose uptake with the expressions of glucose transporter type 1 and Na^+^/I^−^ symporter in patients with untreated papillary thyroid carcinoma	To compare the uptake of FDG-F18 with the expression of GLUT1 and NIS in tumors of patients diagnosed with PTC undergoing curative surgery	2013	Korea	*N* = 33	PET/CT	The SUV in lesions with NIS-negative expressions was higher than that in the lesions with positive expression of NIS.	*P* = 0.006
Kleiman et al. [24]	Thyroid-stimulating hormone increases iodine uptake by thyroid cancer cells during BRAF silencing	To evaluate the combined effect of BRAF inhibition and TSH supplementation on iodine-131 uptake in thyroid cancer cells with BRAFV600E	2013	USA	*N* = 47	qPCR	The expression of NIS in the group of patients with thyroid cancer (of which 86.5% were PTC) with mutation was lower.	*P* < 0.01
Wang et al. [25]	Differential expression of the Na+/I-symporter protein in thyroid cancer and adjacent normal and nodular goiter tissues	To determine whether the NIS protein was differentially expressed in papillary thyroid cancer and several surrounding tissues	2013	China	*N* = 114	Immunohistochemistry	NIS protein was expressed especially in the cytoplasm.	*P* = 0.675
Wei et al. [26]	Low expression of sodium iodide symporter expression in aggressive variants of papillary thyroid carcinoma	To investigate the difference of NIS expression in aggressive variants, including high cell variant (TCV), diffuse sclerosing variant (DSPTC), and conventional PTC	2014	China	*N* = 312	Immunohistochemistry	In all conventional NIS-positive PTCs, they showed strong staining for tissue identification. NIS expression was identified mainly in the membrane and in the nucleus and was also observed in the cytoplasm in some cases of PTC.	*P* = 0.000
Kim et al. [27]	Expression patterns of glucose transporter-1 gene and thyroid specific genes in human papillary thyroid carcinoma	To compare the differentiation of PTC with the expression of the glucose-1 transporter gene (Glut-1), in addition to other thyroid specifics	2014	Korea	*N* = 24	RT-PCR	The NIS expression in patients with well-differentiated cancer was higher compared to that in patients with less-differentiated cancer.	*P* = 0.0001
							Negative correlation between NIS gene expression and Glut-1 in PTC patients (*r* = 0.60)	*P* < 0.01
							Nevertheless, in these patients, there was a positive correlation between NIS and TG expression (*r* = 0.52); NIS and pendrin (r = 0.77)	*P* < 0.05
Choi et al. [28]	B-RafV600E inhibits sodium iodide symporter expression via regulation of DNA methyltransferase 1	To analyze whether NIS expression was correlated with the expression of BRAFV600E as well as the existence of the methylation of the CpG island	2014	Korea	*N* = 30	Immunohistochemistry/qPCR	NIS staining in the plasma membrane region of the normal thyroid follicle and was reduced in PTC BRAFV600E. 37% of PTC tissues presented diffuse cytoplasmic staining.	NA
							NIS expression is downregulated.	NA
							NIS expression was reduced when BRAFV600E was expressed in normal primary thyrocytes isolated from tissues via lentivirus transduction.	NA
							Thyrocytes expressing BRAFV600E do not efficiently absorb the iodide.	*P* < 0.01
							Inhibition of NIS expression by inducing methylation of the CpG island in the NIS promoter region. The expressions DNMT1 and NIS were inversely correlated.	*P* < 0.01
Sponziello et al. [29]	PDE5 expression in human thyroid tumors and effects of PDE5 inhibitors on growth and migration of cancer cells	To analyze the expression of PDE5 in a series of PTC carcinomas with or without BRAFV600E mutation, classified according to the ATA risk criteria	2015	Italy	*N* = 86	qPCR and MTT viability test	Higher levels of PDE5A expression in PTC patients compared with controls. NIS mRNA expression levels were decreased in patients with PTC.	*P* < 0.0001
Rosignolo et al. [30]	Reduced expression of THR*β* in papillary thyroid carcinomas: relationship with BRAF mutation, aggressiveness and miR expression	To analyze the expression of THR*β*, NIS, TPO, Tg, and TSH-R mRNA in PTC surgical samples	2015	Italy	*N* = 36	qPCR	Positive correlation was detected with THR*β* levels and NIS mRNA levels (ra = 0.3771).	*P* = 0.0098
							The levels of NIS expression in tumor tissues are lower than the corresponding normal.	*P* < 0.001
							Mutation in the BRAFV600E gene is associated with decreased NIS expression.	*P* < 0.0001
Ma et al. [31]	FoxP3 in papillary thyroid carcinoma induces NIS repression through activation of the TGF-*β*1/Smad signaling pathway	Hypothesize that FoxP3 can inhibit NIS membrane expression and cleavage by inducing TGF-*β*1 secretion and subsequent activation of the Smad signaling pathway	2016	China	*N* = 90	Immunohistochemistry	NIS staining was located almost exclusively on the plasma membrane in normal thyroid tissues; however, the staining was less intense in tumor tissues.	NA
							The FoxP3-positive group had a lower rate of NIS staining.	*P* < 0.01
						qPCR	NIS transcript levels were significantly reduced in the tumor sample.	*P* < 0.001
							NIS transcript levels decreased and reached the minimum concentration in 10 ng/mL TGF-beta1.	*P* < 0.01
							NIS protein levels were also strongly suppressed by TGF-*β*1, and there was decrease in relation to the control group.	*P* < 0.01
Dong et al. [32]	Effects of BRAF^V600E^ mutation on Na^+^/I^−^ symporter expression in papillary thyroid carcinoma	To discuss the association of BRAFV600E mutation and NIS expression in PTC tissue and the possible implications on iodine therapy	2016	China	*N* = 134	qPCR	The BRAFV600E mutation did not show inhibitory effect on NIS expression.	*P* = 0.085
						Immunohistochemistry	After adjusting for confounding factors, the mutated PTC BRAFV600E showed lower NIS expression than the wild type in the cases without Hashimoto thyroiditis.	*P* = 0.046
Yazgan et al. [33]	The correlation of sodium iodide symporter and BRAFV600E mutation in classical variant papillary thyroid	To correlate the BRAFV600E mutation and the expression of NIS in PTC classical variant patients	2016	Turkey	*N* = 96	qPCR	Functional expression of NIS was greater.	*P* = 0.047
							Nonfunctional expression of NIS was lower.	*P* = 0.047
Stasiołek et al. [34]	The molecular effect of diagnostic absorbed doses from 131I on papillary thyroid cancer cells in vitro	Obtain information on possible cellular and molecular mechanisms underlying the stunning phenomenon	2017	Poland	—	qPCR/K1 cell culture	Expression of the NIS protein in the K1 cells analyzed after 24 h incubation with iodine-131 showed a positive regulation in groups of the lower (5 Gy) and higher (20 Gy) doses of radioactive iodine.	*P* < 0.05
							Statistically significant changes in the 8-oxo-dG level were observed in K1 cells treated with the lowest 131I (5 Gy) dose compared to the control, even 96 h after treatment, indicating a prolonged accumulation of DNA damage even after the end of irradiation with iodine-131.	*P* < 0.01
